# The complete chloroplast genome of *Meconopsis bella* Prain 1894 (Papaveraceae), a high-altitude plant distributed on the Qinghai-Tibet plateau

**DOI:** 10.1080/23802359.2024.2306879

**Published:** 2024-01-25

**Authors:** Yali Ding, Xinzhong Li, Zhuoma Yang, Qi Wu, Wenji Zhao

**Affiliations:** a Department of Science and Technology, Medical School, Tibet University, Lhasa, China; b School of Sciences, Tibet University, Lhasa, China; c Institute of Chinese Herbal Medicines and Flowers, Sichuan Academy of Grassland Sciences, Chengdu, PR China

**Keywords:** *Meconopsis bella*, papaveraceae, chloroplast genome, Qinghai-Tibet plateau

## Abstract

*Meconopsis bella* Prain 1894 (*M. bella*) is a rare herb within the family Papaveraceae of which unique and gorgeous purple flowers are blooming in the flowering phase. In this study, we reported the complete chloroplast (cp) genome of *M. bella*, which was mainly distributed on the Qinghai-Tibet plateau. The complete chloroplast genome sequence of *M. bella* was 153,073 bp in size and was characterized by a typical quadripartite structure consisting of a large single-copy (LSC) region of 83,562 bp, a small single-copy (SSC) region of 178,33 bp and two identical inverted repeats (IR) regions of 25,839 bp. The genome contained 133 genes, including 88 protein-encoding genes, eight ribosomal RNA genes, and 37 transfer RNA genes. Phylogenetic analysis based on the maximum-likelihood (ML) method showed that *M. bella* was closely related to *M. paniculate* and *M. pinnatifolia* within the genus *Meconopsis*.

## Introduction

The genus *Meconopsis*, belonging to the Papaveraceae family of herb angiosperms, comprises approximately 49 species, with a significant presence of about 38 species in China (Wang and Chen 2003). The *Meconopsis* species is a group of high-altitude plants with significant economic value. Due to their characteristics of vibrant flower colors and high ornamental appeal, they have become renowned as rare alpine flowers, both domestically and internationally (Jun-Hua et al. 2011). *M. bella*, belonging to the genus *Meconopsis*, is a rare alpine herbaceous flowering plant widely distributed on the Qinghai-Tibet Plateau. As a perennial flowering plant, it grows from the root stalk as opposed to from seed. This pretty blue poppy thrives in poor, drained soil, and on a cool, half-shaded raised bed. Due to its unique and challenging ecological environment characterized by factors such as hypoxia, low temperatures, and intense solar radiation, its adaptive strategies have captured significant interest among scientists. However, the lack of genomic resources has severely limited this research.

**Figure 1.**
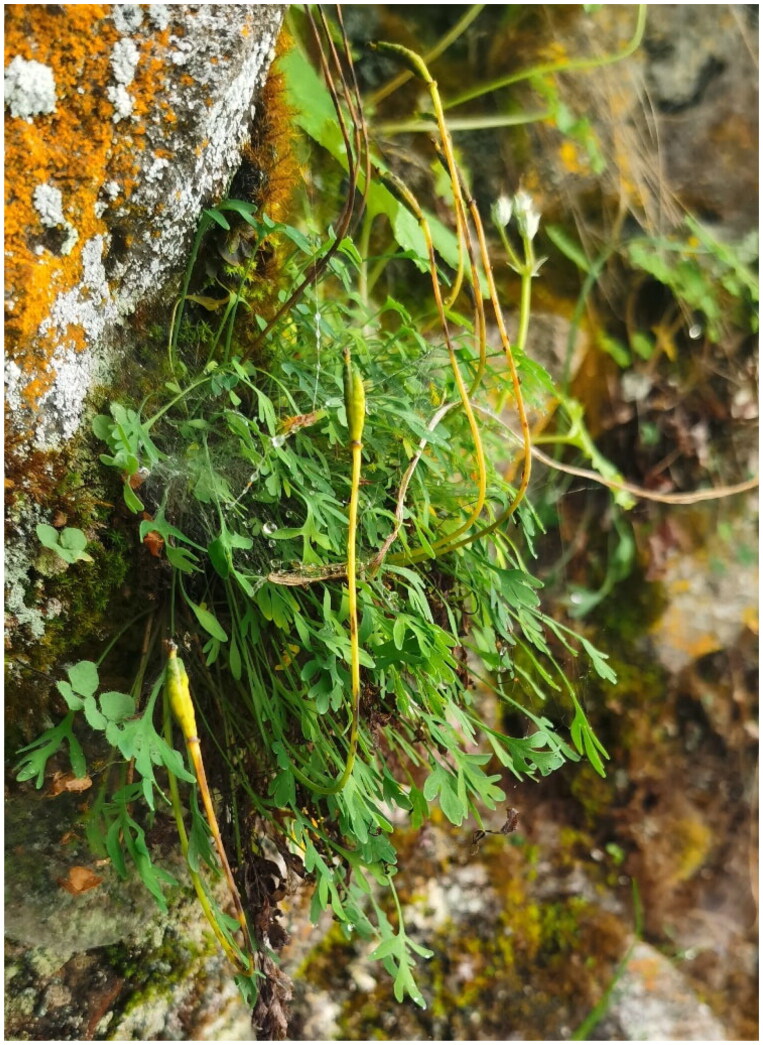
Plant image of *Meconopsis bella*. This species is characterized by a thick taproot with old petioles. There are no leaves on the stems, only the base. The basal leaves may be whole or lobed. The flowers are nodding, and grow singly on slender stems. This photo was taken by author Xinzhong Li at Gilon County in Sichuan Province, with the author’s approval for use. The plant was collected from Gilon County in Sichuan Province, and aerial parts of the plant, including young leaves, were captured in the photo.

As the most crucial photosynthesis organelle, chloroplast performs significant physiological functions. The complete genome within the chloroplast is also a valuable resource for photosynthesis and extreme environment adaptation studies, molecular identification, and phylogenetic evidence (Yu et al. 2020; Zhao et al. 2020; Li et al. 2021; Ma et al. 2021; Yu et al. 2023). Therefore, we sequenced and assembled the complete chloroplast genome (cp) genome of *M. bella* (Figure 1), which was collected from the Qinghai-Tibet plateau at an altitude of 3600 meters. This study was expected to enrich the genomic resources of *Meconopsis* species.

## Materials and methods

### Plant material collection and DNA extraction

The fresh leaves were collected from a single individual from Gilon County, Tibet, China (85.41° E, 28.40° N) and deposited immediately in liquid Nitrogen. The voucher specimens were deposited in the State Key Laboratory of Hybrid Rice, College of Life Sciences, Wuhan University (http://sklhr.whu.edu.cn/, Xing Liu, xingliu@whu.edu.cn), under the voucher number MHGL2022. Total genomic DNA was extracted according to the standard protocol of the Genomic DNA extraction kit (TIANGEN, China).

### Sequencing, assembling, and annotating the chloroplast genome

The 2 × 150 bp library was constructed and sequenced with an average insert size of 350 bp on the BGISEQ-500 platform. The cp genome was assembled into a circular contig with GetOrganelle v1.6.1a (Jin et al. 2020) with k-mers set to 75, 95, 115, and 127, along with other default parameters. Then, the cp genome was confirmed by mapping the raw reads using BWA v0.7.17 (Li and Durbin 2009), pilon v1.24 (Walker et al. 2014), and bowtie2 v2.4.4 (Langmead and Salzberg 2012). The reads coverage plot is pictured using Geneious v11.0.5. Cp genome annotation was performed using both GeSeq (Tillich et al. 2017) and CPGAVAS2 (Shi et al. 2019) with the default settings, which regarded the *M. racemosa* (NC_039625) as a reference. Then, the result was corrected manually for start and stop codons and for intron/exon boundaries. The gene graphical map of the chloroplast genome was constructed using cpgview (http://www.1kmpg.cn/cpgview) (Liu et al. 2023). The complete cp genome sequence together with gene annotations were submitted to GenBank under the accession number OR030860.

### Phylogenetic analysis

To better understand the phylogenetic relationship of *M. bella* in this study, *M. bella* and other 27 cp genomes of *Meconopsis* genus available in GenBank were used to perform phylogenetic analysis, along with two cp genomes in genus *Papaver* as outgroups. The 88 shared protein-coding genes were extracted, aligned separately, and concatenated to construct a matrix using PhyloSuite_v1.1.15 (Zhang et al. 2020). ML analyses were inferred using IQ-TREE under the model automatically selected by IQ-TREE (Nguyen et al. 2015).

## Results and discussion

**Figure 2.**
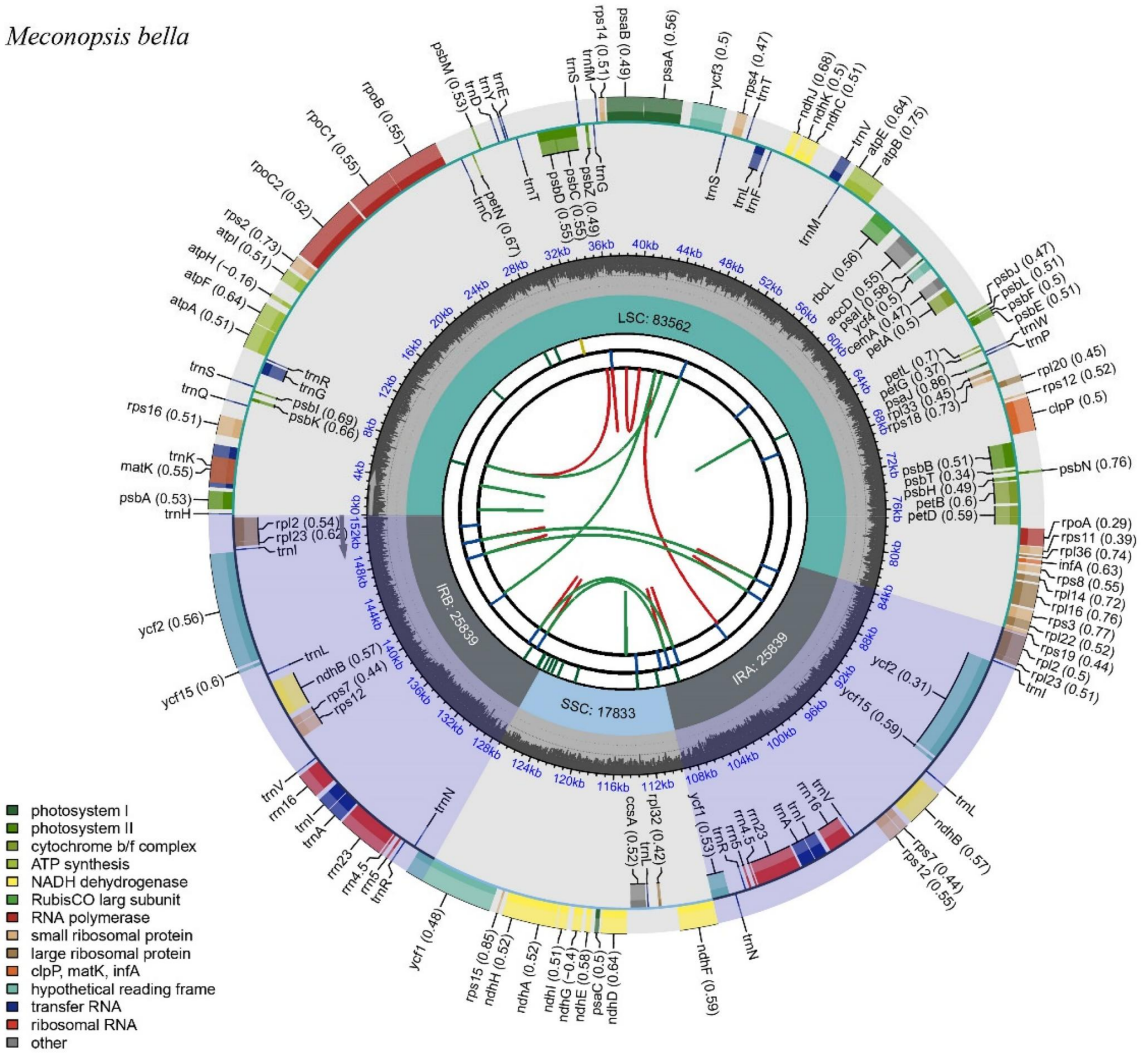
Chloroplast genome map of *M. bella*. Genes inside the circle are transcribed clockwise, while those outside are transcribed counterclockwise. Genes are color-coded according to functional groups. The dark pink region inside the inner circle indicates the GC content, while the green color indicates the at content of the cp genome. Boundaries of the small single copy (SSC) and large single copy (LSC) regions and inverted repeat (IRa and IRb) regions are denoted in the inner circle.

**Figure 3.**
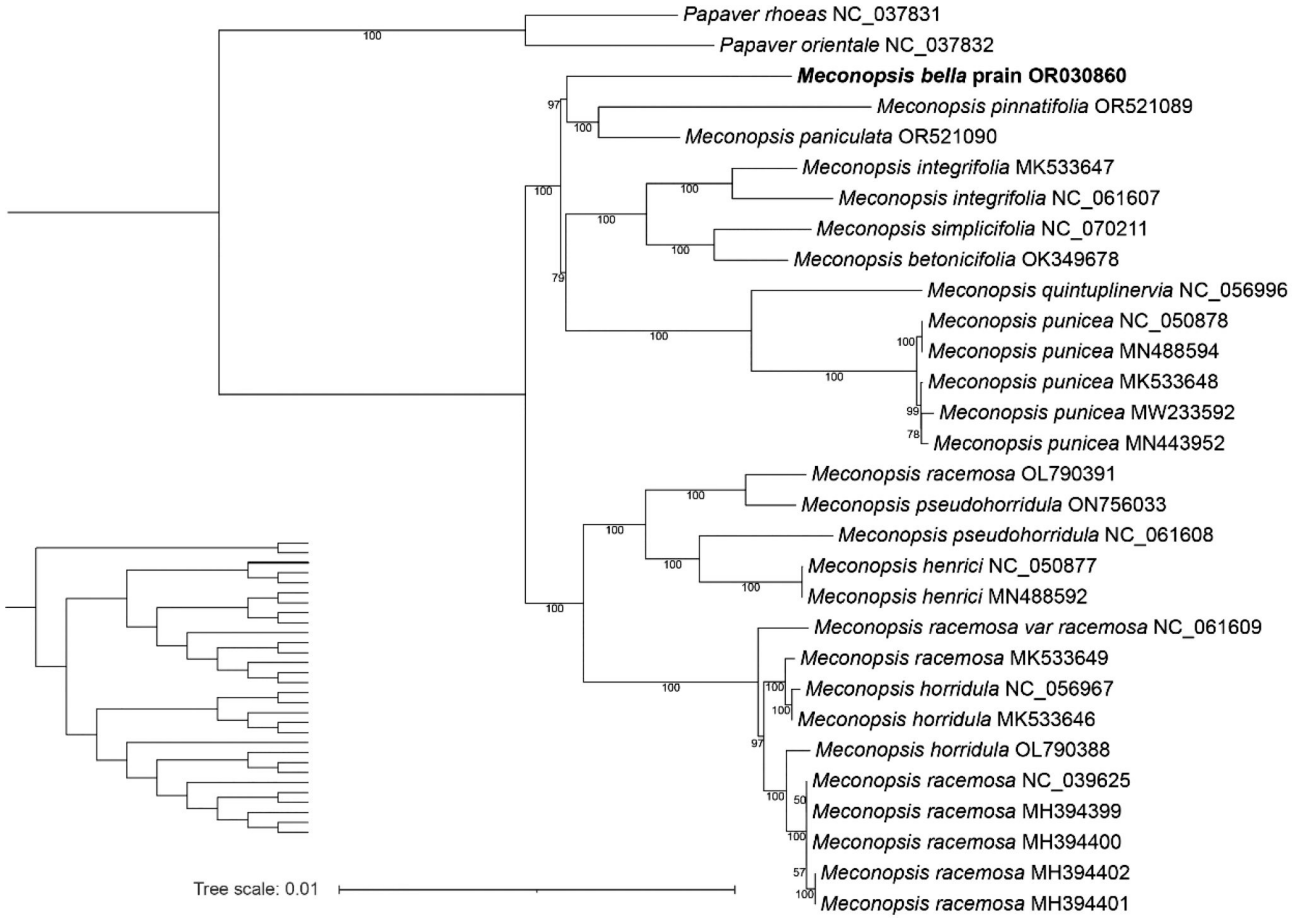
Maximum likelihood phylogenetic tree of *M. bella* and other 29 plant species constructed using their shared 88 protein-coding nucleotide sequences of cp genomes. The best-fit model according to AIC: TVM + F + I + G4. The bootstrap support value for each node is shown on the branch. In the lower left corner of the diagram is placed an cladogram showing a clear topology without species names. The accession number of the cp genome of each plant species is shown in the brackets. The following sequences were used: *Papaver rhoeas* NC_037831 and *Papaver Orientale* NC_037832 (Zhou et al. 2018), *M. racemosa* NC_039625 (Zeng et al. 2018), *M. horridula* MK533646, *M. integrifolia* MK533647 (Li et al. 2019), *M. pseudohorridula* NC_061608 (unpublished), *M. henrici* NC_050877 and *M. punicea* NC_050878 (Zhu and Zhang 2019), *M. simplicifolia* NC_070211 (unpublished), *M. betonicifolia* OK349678 (unpublished), *M. pinnatifolia* OR521089 (unpublished), *M. paniculata* OR521090 (unpublished), *M. integrifolia* NC_061607 (unpublished), *M. quintuplinervia* NC_056996 (Xu et al. 2019), *M. punicea* MN488594 (unpublished), *M. punicea* MK533648 (unpublished), *M. punicea* MW233592 (Ruifang et al. 2021), *M. punicea* MN443952 (unpublished), *M. racemose* OL790391 (unpublished), *M. pseudohorridula* ON756033 (unpublished), *M. pseudohorridula* NC_061608 (unpublished), *M. henrici* MN488592 (unpublished), *M. acemose* var acemose NC_061609 (unpublished), *M. racemose* MK533649 (unpublished), *M. horridula* NC_056967 (unpublished), *M. horridula* MK533646 (Li et al. 2019), *M. horridula* OL790388 (unpublished), *M. acemose* NC_039625 (Zeng et al. 2018), *M. racemose* (MH394399-MH394402) (Zeng et al. 2018).

The analysis of the complete cp genome of *M. bella* sheds light on various aspects of its genomic structure and gene content. The high throughput DNA sequencing reads mapping to the cp genome sequences with bowtie2 is applied as a genome structure verification step, and the mean read coverage is 1237× (Figure S1). The results show that the coverage is relatively uniform, indicating that the cp genome is assembled correctly (Figure S1). The structure of the genes that are difficult to annotate is also provided in Figure S2 (cis-splicing gene map) and Figure S3 (trans-splicing gene map), which demonstrates the correctness of our genome annotation. The complete cp genome of *M. bella* is 153,073 bp in length, including a LSC of 83,562 bp and a SSC of 17,833 bp separated by a pair of identical IRs of 25,839 bp (Figure 2). The size of the *M. bella* cp genome (153,073 bp) falls within the typical range observed in angiosperms, further supporting the representation of the species within the larger group of flowering plants (Daniell et al. 2016). The GC content of the whole genome, IRs, LSC, and SSC regions were 43.2%, 37.5%, and 33.5%, respectively. In addition, a total of 133 genes were annotated, comprising 88 protein-coding genes, 8 rRNA genes, and 37 tRNA genes. The annotation of a total of 133 genes in the *M. bella* cp genome provides valuable insights into its functional repertoire. The 88 protein-coding genes identified in this study encode proteins responsible for various essential functions, including photosynthesis, transcription, translation, and energy metabolism. The gene content of the *M. bella* cp genome is highly consistent with other plant species within the Papaveraceae family and such conserved gene content supports the evolutionary conservation of the Papaveraceae family group (Li et al. 2019). As shown in Figure 3, the *M. bella* collected in this study forms a monophyletic clade with the Papaveraceae family and is closely related to *M. pinnatifolia* and *M. paniculata*. Phylogenetic analysis using the complete cp genome sequence can elucidate the evolutionary relationships between *M. bella* and other closely related species, thus our results contribute to a better understanding of the evolutionary history of the Papaveraceae family.

## Conclusion

In conclusion, we have successfully characterized the complete cp genome of *M. bella*, revealing its genomic structure, GC content variations, and gene content. These results contribute to the understanding of cp genome evolution and provide a valuable resource for further studies in plant phylogenetics, taxonomy, and conservation biology. The findings presented here underscore the significance of cp genomes as powerful tools for plant genetic research and applications in various disciplines of botanical science.

## Ethical approval

Research and collection of plant material was conducted according to the guidelines provided by Tibet University. Permission was granted by the Local Development Funds of Science and Technology Department of Tibet.

## Supplementary Material

Supplemental Material

## Data Availability

The genome sequence data that support the findings of this study are openly available in GenBank with accession number (OR030860) (http://www.ncbi.nlm.nih.gov/). Raw sequencing reads used in this study have been deposited in the SRA database of NCBI under accession number SRR25582714. The associated “BioProject,” and “Bio-Sample” numbers are PRJNA1003862, and SAMN36908543 respectively.
