# Supplementary material for: The complete chloroplast genome of Meconopsis bella Prain 1894 (Papaveraceae), a high-altitude plant distributed on the Qinghai-Tibet plateau
Source: Mitochondrial DNA B Resour. 2024 Jan 25;9(1):195–9. doi: 10.1080/23802359.2024.2306879 (PMC10812850; doi:10.1080/23802359.2024.2306879)
Supplement: Supplemental Material [file TMDN_A_2306879_SM2595.docx]

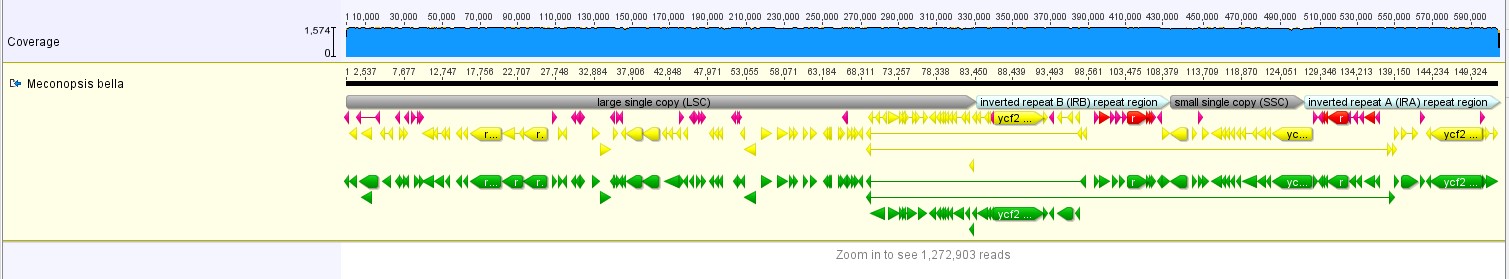


Figure S1. Coverage of chloroplast genome sequencing based on short reads. The upper part of the figure shows the coverage in blue, and the lower part shows the structure of the chloroplast genome, in which the green ones are genes, the yellow ones are protein-coding genes, the pink ones are tRNA, and the dark red ones are rRNA.


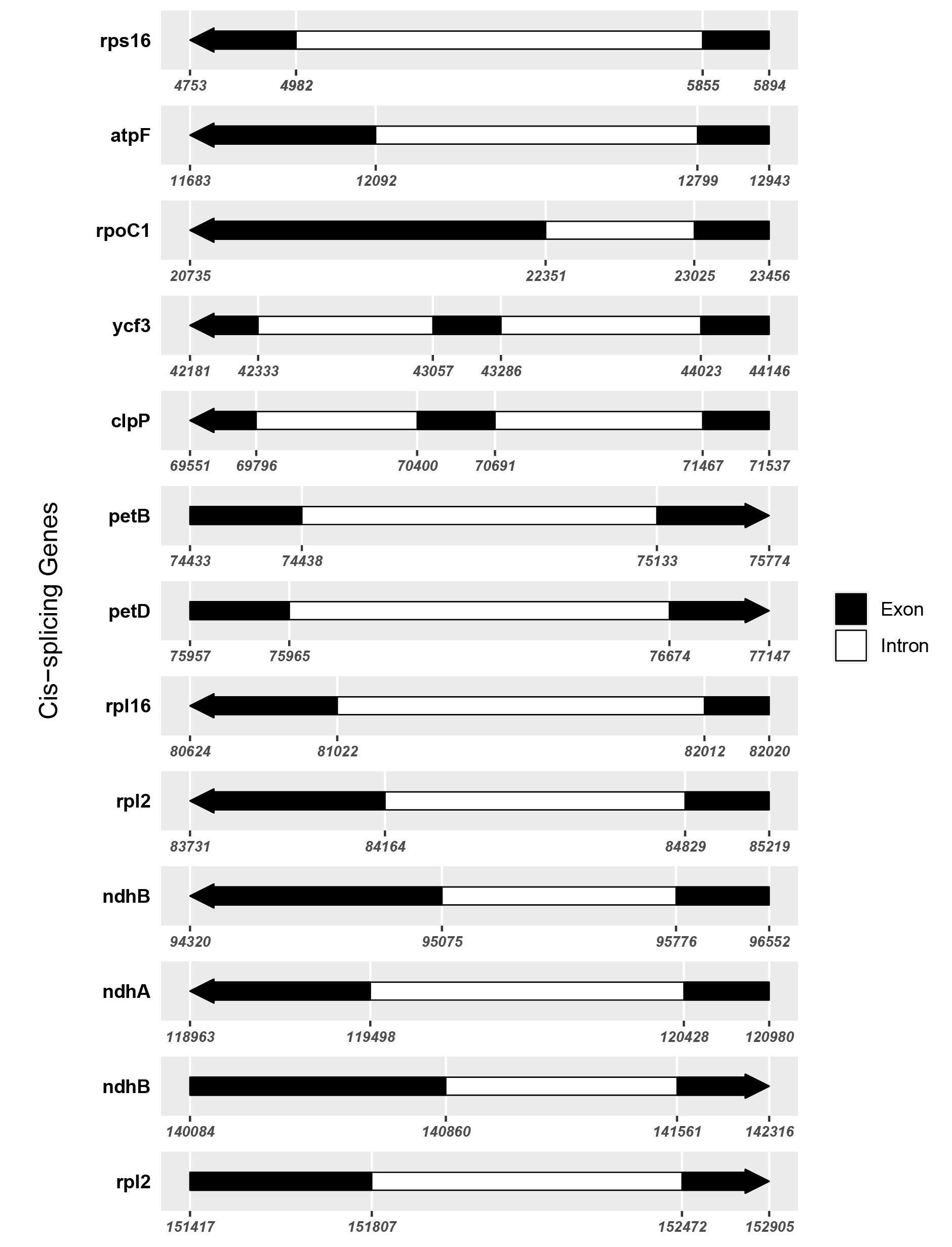


Figure S2. Schematic maps of cis-splicing genes in the chloroplast genome of *M. bella*. Maps generated using CPGView. The gene names are shown on the left, and the gene structures are on the right.


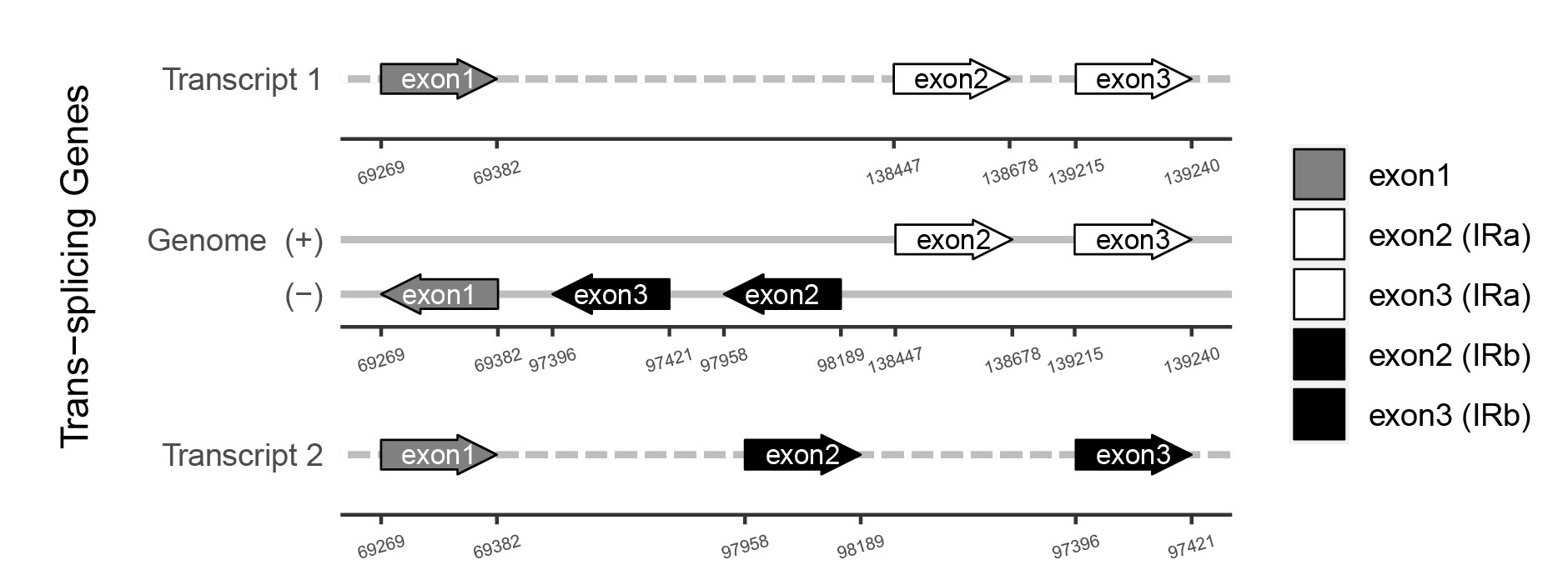


Figure S3. Schematic maps of the trans-spliced gene *rps1*.
